# Amalgamation of PI3K and EZH2 blockade synergistically regulates invasion and angiogenesis: combination therapy for glioblastoma multiforme

**DOI:** 10.18632/oncotarget.27842

**Published:** 2020-12-22

**Authors:** Vishnu S. Mishra, Naveen Kumar, Masoom Raza, Seema Sehrawat

**Affiliations:** ^1^Precision NeuroOncology & NeuroVascular Disease Modeling Group, Department of Life Sciences, School of Natural Sciences, Shiv Nadar University, NCR 201314, India; ^*^These authors contributed equally to this work

**Keywords:** precision neuroOncology, combination therapy, glioblastoma multiforme, pharmacological intervention, cytokine based proteomics

## Abstract

Glioblastoma multiforme is known as the primary malignant and most devastating form of tumor in central nervous system of adult population. Amongst all CNS cancers, Glioblastoma multiforme GBM is a rare grade IV astrocytoma and it has the worst prognosis initiated by metastasis to supra-tentorial region of the brain. Current options for the treatment include surgery, radiation therapy and chemotherapy. Substantial information of its pathology and molecular signaling exposed new avenues for generating innovative therapies. In our study, we have undertaken a novel combination approach for GBM treatment. PI3K signaling participates in cancer progression and plays a significant role in metastasis. Here, we are targeting PI3K signaling pathways in glioblastoma along with EZH2, a known transcriptional regulator. We found that targeting transcriptional regulator EZH2 and PI3K affect cellular migration and morphological changes. These changes in signatory activities of cancerous cells led to inhibit its progression *in vitro*. With further analysis we confirmed the angiogenic inhibition and reduction in stem-ness potential of GBM. Later, cytokine proteome array analysis revealed several participants of metastasis and tumor induced angiogenesis using combination regime. This study provides a significant reduction in GBM progression investigated using Glioblastoma Multiforme U-87 cells with effective combination of pharmacological inhibitors PI-103 and EPZ-6438. This strategy will be further used to combat GBM more innovatively along with the existing therapies.

## INTRODUCTION

Aggressive metastatic tumor is the leading cause of cancer death in patients worldwide. Cancer accounted for approximately 9.6 million deaths in 2018 and statistically out of 6 there is 1 death due to cancer, globally [1] as per World Health Organization. Glioblastoma is known as the most frequent tumor in brain and aggressive in all human cancers [2, 3]. Regardless of these statistics, the brain tumor derived Glioblastoma needs utmost care towards therapeutic intervention due to limited therapeutic courses. Therapy limitations are the major cause of limited survival in patients approximately 10–14 months. Leaky and rich vasculature of GBM (U-87) is one of the most important cause of its aggression [4]. Due to higher rate of leakiness and VEGF secretion by GBM tumor cells, anti-VEGF therapy or anti-angiogenesis therapy (Avastin) is being utilized in clinical trials. A combination therapy, involved with bevacizumab and irinotecan, used in phase II clinical trial affect GBM in 50% population of patients but the effect was transient [5, 6]. In current situation, GBM treatment involves surgical removal and radiation therapy with adjuvant Temozolomide [2].

Glioblastoma multiforme is associated with fatal outcomes even during and after treatment. Heterogeneity of tumors is the prime cause of resistance and recurrence. Local recurrence represents immediate fatality in GBM due to chemotherapy and radiotherapy resistance [7–9]. Blood brain barrier presence hinders the therapeutic potential of chemotherapeutics and gene therapy in most of the cases. Glioblastoma multiforme is associated with high expression of CD24 and ROS at basal level [10–12]. Many studies reported that aggressiveness of Glioblastoma is linked with CD24 and ROS generation. Interestingly, ROS basal level expression is very high in GBM cells [13].

Despite, the current treatment including chemotherapy, surgery and radiotherapy the overall survival rate is less than 2–3 years for GBM patients [14, 15]. Recent advances for GBM treatment include hormonal and combinational approach and it allows cohort examination along with other therapies of the patients. As therapy failure gives the chance to innovate new approaches, it is necessary to elucidate novel regimes for the patients. This will give a vast data set of novel combinations of drugs which can be further utilized as a personalized medicine for different patients.

We are reporting a novel combination regime for the inhibition of GBM aggression in *in vitro* conditions. In our studies, we worked on a combination that involved PI-103 and EPZ-6438 to treat GBM. Our aim was to target two separate but major signaling pathways in GBM cell cycle progression. Here, we focused on PI3K and EZH2 signaling in GBM cells. PI3K works as a signal transducer enzyme for cell proliferation and intracellular trafficking in GBM. Cellular growth and cellular proliferation are directly linked with cancer cell progression. GBM showed a high range of mutation in PI3K subunit p110α and thus it is more active and responsible for tumor progression [16, 17]. On the other hand, we focused on a separate signaling of EZH2, which is known as transcriptional repressor. The basic target of EZH2 is histone methylation that causes transcriptional repression in general. EZH2 functions to inhibit tumor suppressor genes in many cancer tissues including GBM [18–21]. GBM cells shows a healthy amount of EZH2 expression and thus cause high malignancy. A specific inhibitor of EZH2 can reduce its expression and halt the cell growth.

We are highlighting the synergistic effect of our novel targeting approaches in GBM treatment using Glioblastoma Multiforme U-87 cells as the model system. We are presenting a significant reduction of GBM progression while targeting with PI-103 and EPZ-6438. Our outcomes showed that the combination regime inhibits the cells at sub G1 phase and reduces the ROS level initially. PI-103 acts as a major player but many results suggested that EPZ-6438 combination adds new dimensions to the effect of PI-103. Rigorous therapies alter the cells basic structure and also helps in generation of a small subset of stem cell populations, which causes the re-occurrence of GBM in patients after heavy load of therapies. Interestingly, we observed a significant inhibition of GBM stem-ness property during a two-week treatment of PI-103 and EPZ-6438 combination. Later we performed a cytokine profiling proteome array to investigate many molecules that can be targeted by inhibiting PI-103 and EPZ-6438 combination treatment. We found a diverse group of molecules which were either directly or indirectly participating in GBM progression and their expression was highly modulated in our combination regime. Our study provides a novel precision targeting approach in GBM specifically targeting different signaling pathways which are responsible for GBM progression.

## RESULTS

### PI-103 and EPZ-6438 combination targets GBM progression via precisely modulating cytoskeleton reorganization and reduced adhesion

**Figure 1:**
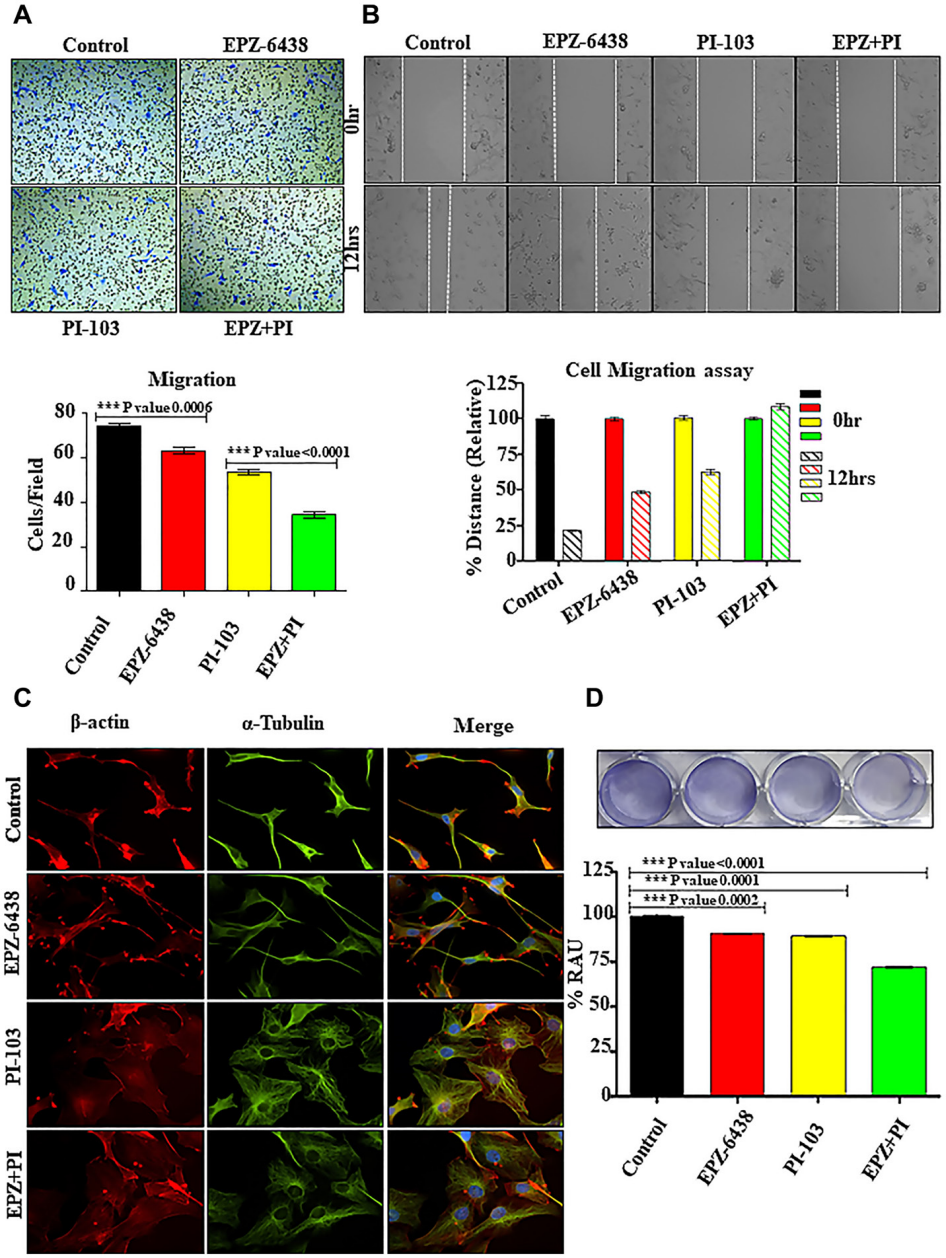
EPZ-6438 and PI-103 hinders the cellular migration of GBM U-87 cells. (**A**) Boyden chamber analysis was performed for cell migration properties. Combination of drugs shows that a smaller number of migrated cells compared to control. (**B**) Wound healing assay shows the similar pattern of migration inhibition during combination of PI-103 & EPZ-6438. (**C**) Cytoskeleton analysis depicted the deregulation of actin and tubulin after treatment. It accounts for the morphological changes in the cellular architecture after the treatment EPZ and PI-103. (**D**) Adhesion properties of GBM U-87 cells was altered during treatment of combination of drugs and is one of the reasons of reduced cell migration during treatment.

GBM U-87 cells have the tendency to migrate exponentially in microenvironment conditions. PI-103 and EPZ-6438 drugs were tested for targeting GBM U-87 progression. PI-103 and EPZ-6438 have different targets and signaling pathways, hence lesser opportunity for cross-talk exist. As the available literature lacks the information regarding the safe number of drugs, counting assay was used to determine the IC50 values (Supplementary Figure 1A) for further use. We have also found the effect of EPZ-6438 and PI-103 on HEK-293, PC3 and MDA-MB-231 cells for comparative analysis with GBM U-87 cells (Supplementary Figure 1B). Combination of drug molecules specially reduced the migration in Boyden chamber analysis. Control cells shows the high number of migrated cells which is also confirmed with 2D wound healing analysis (Figure 1A and 1B). GBM U-87 migratory properties are responsible for its aggression and fatality. Tumor cell migration is profoundly reliant on morphological changes, associated with vigorous changes in actin. Cell motility is the result of rearrangement of cytoskeleton and it helps to move cells towards forward directions [22]. Tubulin and actin reorganization showed the irregular shape of GBM U-87 cells during combination treatment and also reduced adhesion leads to inhibition of cell migration (Figure 1C and 1D). We have already discussed in our results that this behavior of cell motility is associated with adhesion properties, cytoskeleton reorganization and/or cell cycle properties. Loss of adhesion during cellular treatment is one of the profound reasons for decreased migration.

### PI-103 and EPZ-6438 combination block the cells in G-S transition without inducing apoptosis

**Figure 2:**
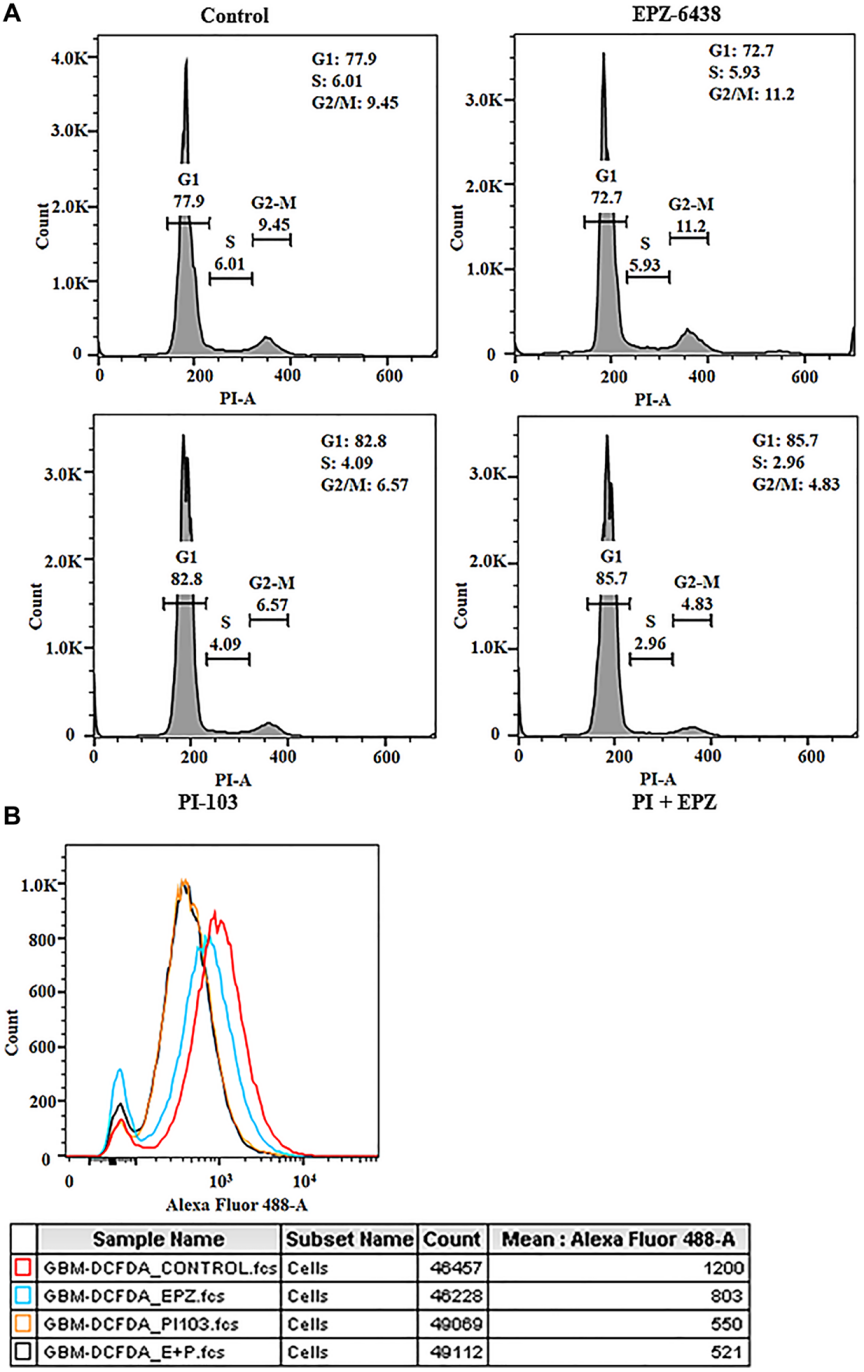
EPZ-6438 and PI-103 regulates the cell cycle progression and ROS generation. (**A**) Cell cycle analysis was conducted to confirm the effect of EPZ and PI-103 on cell migration. Cells were arrested in G1 phase (85.7%) after the treatment with combination of drugs. (**B**) Cell apoptotic analysis was performed to get the insight into the effect of drug. DCFDA analysis showed the reduction of ROS in GBM U-87 cells. GBM U-87 cells have the high frequency of ROS at basal level. This data suggests that GBM U-87 cell progression is reduced after reduction in ROS. This data showed that cells are stuck in G1 phase but not going under apoptosis at this concentration.

As GBM U-87 cells tend to proliferate, combination of drugs was used for cell cycle analysis. Cells were stuck in G to S transition in treated cells compared to control, which laid the foundation of progression inhibition (Figure 2A). Cells blockade in G-S transition does not confirm that cells are undergoing apoptosis. We evaluated the ROS generation and mitochondrial membrane potential (data not shown) in GBM U-87 cells. High basal level of ROS determines its aggression, interestingly we found ROS decreased in combination as well as individual treatment (Figure 2B). It opened a new window for further analysis of mitochondrial membrane potential as it is one of the widely known factor for ROS generation. This data shows that combination treatment although blocked cells in G-S transition but fails to induce apoptosis at these concentrations.

### GBM U-87 cells invasive potential is precisely compromised by EPZ-6438 and PI-103 treatment in combination

**Figure 3:**
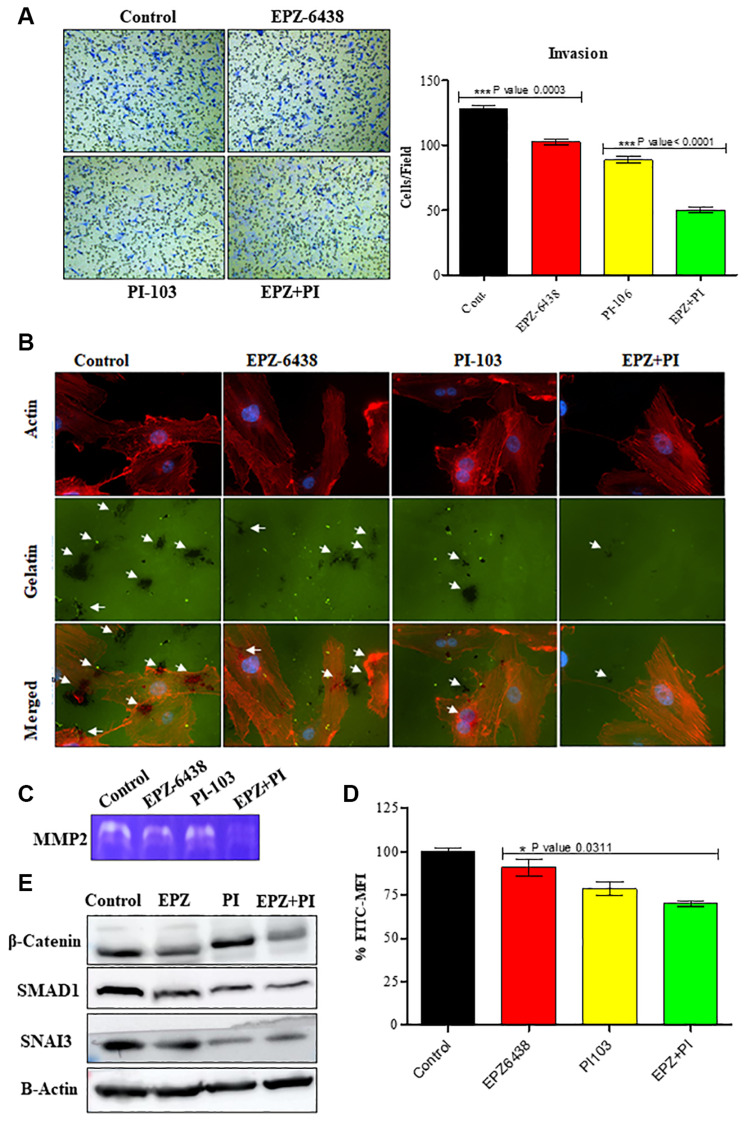
EPZ-6438 and PI-103 reduces the invasive behavior of GBM U-87 cells. (**A**) Invasion assay was performed to analyze the effect of drug molecules on GBM U-87 cells. We found that invaded cells were less compared to the control cells as confirmed with Gelatin degradation assay. (**B**) Small patches showed the gelatin degradation in control and treated cells. We found that the number of invaded patches were less in combination of EPZ and PI-103 treated cells. (**C**) Invaded cells activity was also analyzed with MMP2 assay. We found that MMP -2 expression reduced while treating the cells. MMP-2 reduction suggests the inability of GBM (U-87) cells to invade the surrounding tissue. (**D**) CD24 expression represents the aggression of GBM U-87 cells. We found that combination of EPZ-6438 and PI-103 reduced the expression of CD24. Less expression of CD24 indicates higher reduction in metastasis. (**E**) Western blot analysis performed and showed effective reduction in EMT specific markers. Inhibition of β-catenin along with SMAD1 and SNAI3 showed the reduction of metastatic potential of GBM U-87 cells.

Perivascular space is the desired mode of invasion in GBM U87 cells. It is associated with almost all the Extra Cellular Matrix proteins and causes GBM U-87 infiltration during treatment [23]. GBM U-87 invasion is a collective effort of GBM U-87 cells to interact with ECM and navigate to the surrounding healthy tissue. Transwell migration studies performed with matrigel showed a progressive inhibition in combination treated GBM U-87 cells. Number of invaded cells in transwell membrane were few as compared to the control cells. A significant reduction in individual treatment also showed the potential of combination therapy (Figure 3A). Gelatin degradation assay described the GBM U-87 invasion through multiple black punctate formation in control cells. Control cells degrade the gelatin similarly to ECM degradation in the *in vivo* conditions. Combination data showed very less punctate formation and led to a reduced invasive capability of GBM U-87 cells (Figure 3B). Degrading area was quantified by Image J (Supplementary Figure 2A). ECM degradation is at the high priority during metastasis in cancer progression. MMP 2 actively participate in ECM degradation in GBM U-87 cells, and we have shown through Zymography that MMP2 secretion is inhibited during combination treatment of PI-103 and EPZ-6438 (Figure 3C). MMP2 and MMP9 are categorized as the degraders of extracellular matrix and basement membrane [24]. These activities of MMPs allow the cancer cells to invade and spread to distant organs. Glioma cells express high level of MMP2 which is responsible for its aggression and invasion capabilities [25]. We did not detect MMP9 during the treatment. MMP2 inhibition may explain that cells lose the invasive capabilities after the treatment of combination of drugs. Combination of EPZ-6438 and PI-103 is targeting cellular invasion and metastatic potential of GBM U-87 cells. To further confirm the effect of our combination on GBM U-87 cells metastatic potential, we conducted western analysis for specific EMT markers i.e., β-catenin, SNAIL3, SMAD1. β-catenin has a vital role in cellular homeostasis. Higher expression of β-catenin always acts as an inducer of tumor proliferation and EMT effector. Targeting β-catenin can suppress the tumor cell aggression and we found the expression of β-catenin in combination was reduced [26]. Along with this we also performed a western analysis for SNAIL3 and SMAD1 [27–29]. Combination regime effectively targets both the markers and strengthen our data for inhibition of metastasis potential in GBM cells (Figure 3E). All the blots were quantified and graphs were made (Supplementary Figure 2B). Interestingly, we also analyzed the CD24 expression as it became a major marker for GBM U-87 motility and invasion. CD24 expression gradually reduced in separate and combination treatment of GBM U-87 cells (Figure 3D).

### Combination regime reduces the recurrence properties of GBM U-87 cells

**Figure 4:**
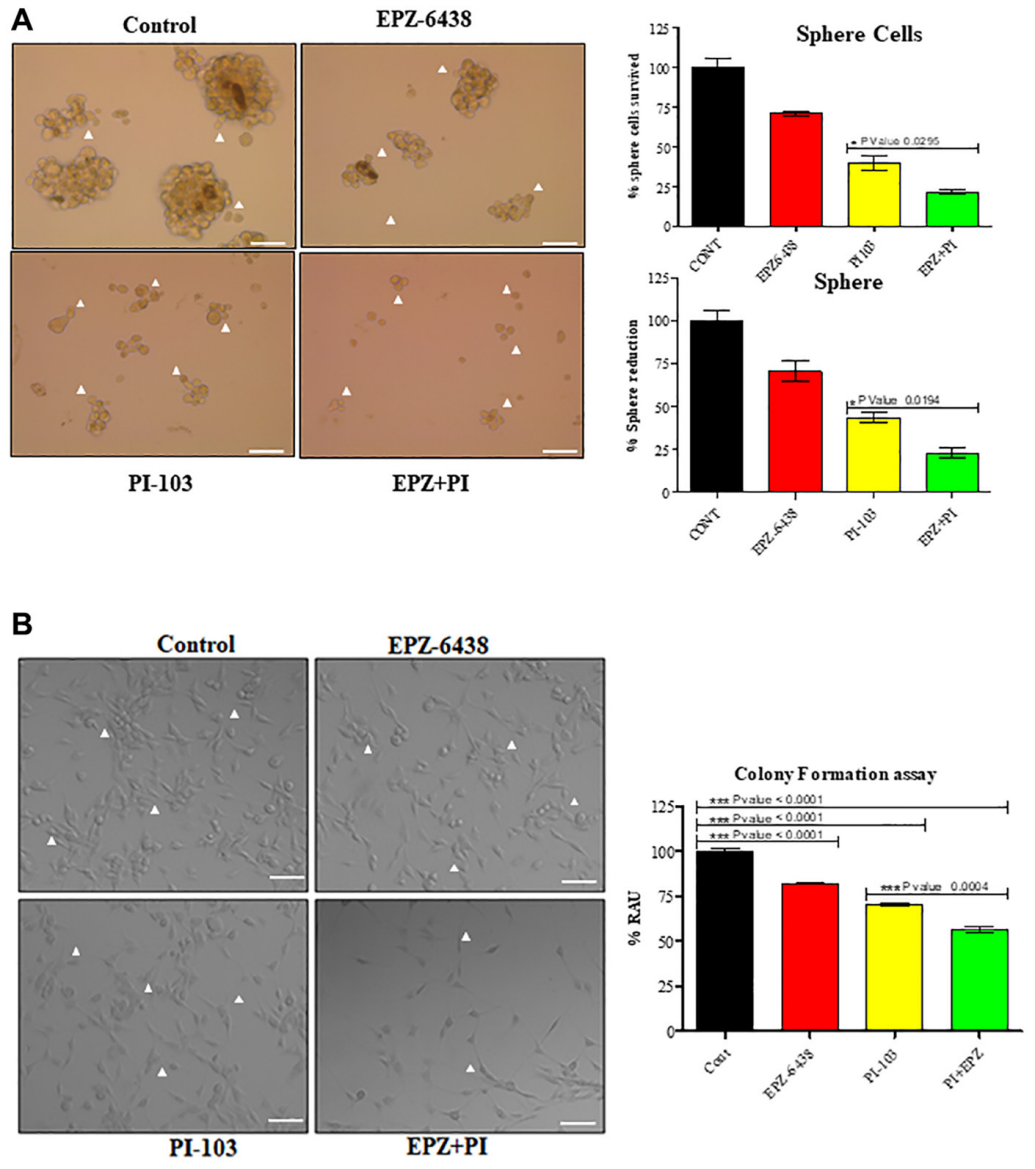
EPZ-6438 and PI-103 combination affect the stemness properties of GBM U-87 cells. (**A**) Mammosphere assay was conducted for stemness properties. Two weeks analysis showed a high reduction in spheroid formation. We found that combination of EPZ and PI-103 reduces the effective stemness properties which promotes spheroid formation and sphere cells *in-vitro*. (**B**) To get the further role of treatment in GBM U-87 cells we performed colony forming assay. Clusters of cells during treatment highly reduced and cells were segregated. Treated cells were not able to make colonies for 2 weeks. Scale bar represents 100 μM.

GBM U-87 recurrence is one of the responsible factors of poor patient prognosis and therapy failure. GBM U-87 contain a very small subset of stem cells (GSCs) that can regrow as a new tumor mass after the therapy [30–32]. Stemness potential of such GSCs is very high. We investigated this property of GSCs through mammosphere studies. Continuous treatment of PI-103 and EPZ-6438 was given after every 3rd day. Data was collected simultaneously under the microscope. Spheroid forming capacity of GBM U-87 cells gradually reduced day by day (Figure 4A). Number of spheroid and sphere cells were very less in combination treatment. This data gives insight about the reduced stemness properties of GBM U87. We have also included another representative image in (Supplementary Figure 3). Although mammosphere formation gave us the idea of inhibition of cell recurrence, we performed 2D colony forming assay for 12 days. Combination of PI-103 and EPZ-6438 reduced the colony formation in GBM U-87 cells (Figure 4B).

### Precision NeuroOncology based effect on GBM U-87 cell metastasis and tumor induced angiogenesis mediated by combination therapy provided by PI-103 and EPZ-6438

**Figure 5:**
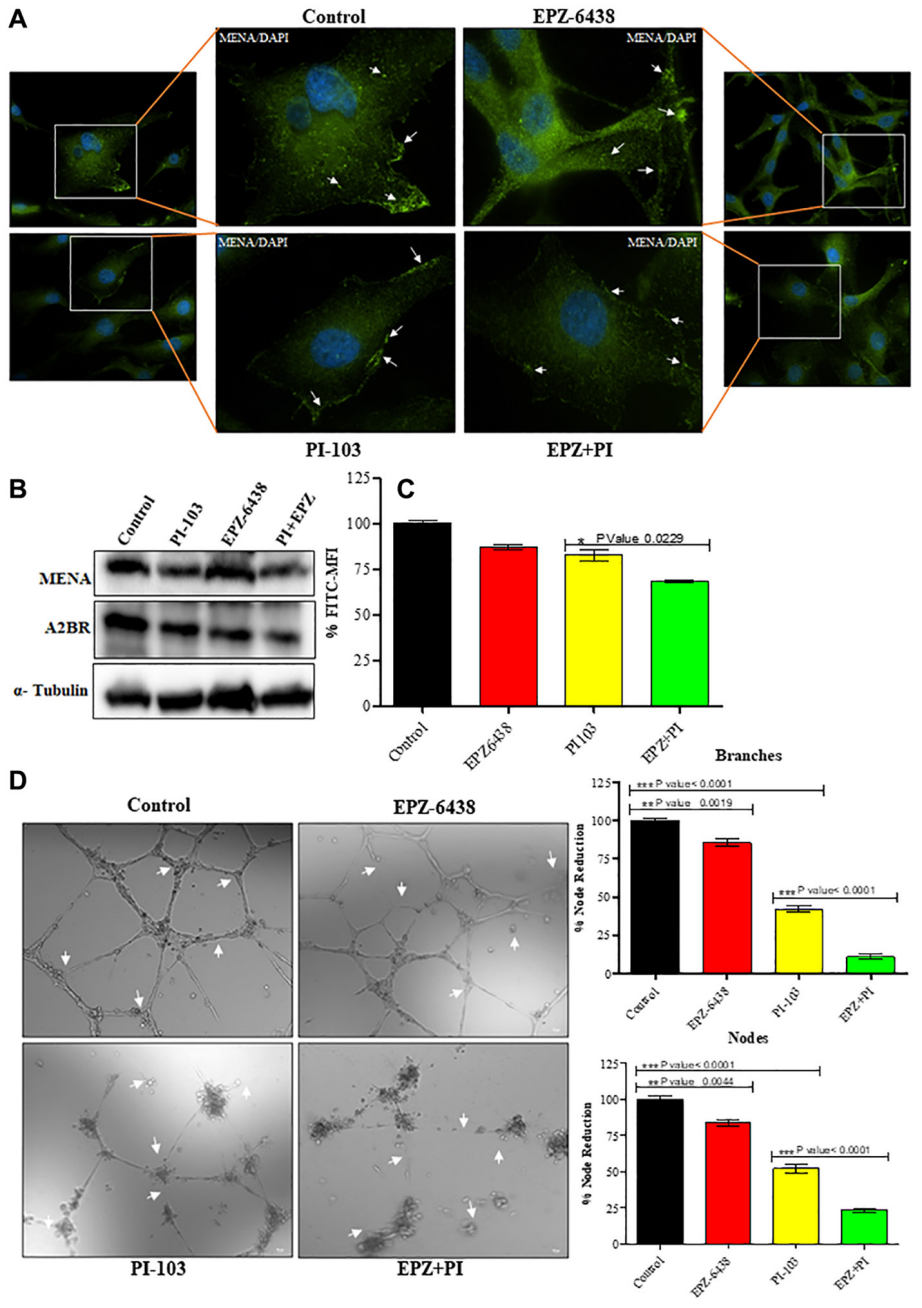
Treatment of EPZ and PI-103 inhibits the angiogenesis. (**A** and **B**) IFA and WB data showed that metastatic markers are altered during combination treatment. Which strongly support that metastasis inhibition during treatment of combination of drugs. (**C**) High level of Ca+2 helps in GBM (U-87) proliferation and stemness potential. We treated the cells with EPZ and PI-103 and found a significant reduction of Ca+2 levels. (**D**) Tube formation assay was performed for GBM (U-87) induced angiogenesis. We found that TCM of treated GBM U-87 cells showed a smaller number of branches sand tubes compare to alone and control cells. This data suggests the role of combined treatment of EPZ and PI-103 in tumor microenvironment. Graphical representation shows the nodes and branches details.

Tumor cells spread mainly in two stages namely distant metastasis and local intra-organ invasion. GBM U-87 metastasize in brain through CSF [33, 34]. Combination treatment of PI-103 and EPZ-6438 was analyzed for tumor metastatic properties using MENA, an actin-modulating protein [35] known for its association with metastasis. We found the downregulation of MENA and A2BR after treatment with combination of the drugs, which causes inhibition of migration and metastasis (Figure 5A and 5B). Interestingly, it is widely known that calcium participates in promotion of many characteristics of cancer [36, 37] and we found a significant reduction in intracellular calcium expression. This regulation in expression supports the idea of cancer inhibition in GBM U-87 cells (Figure 5C). GBM U-87 is rich in vasculature and is characterized by highly angiogenic capability. High proliferation rate causes nutrition depletion in tumor microenvironment. GBM U-87 cells release specific growth factors to induce pre-existing capillaries for neo-angiogenesis. We analyzed the effect of PI-103 and EPZ-6438 on tumor induced angiogenesis. Tissue culture media (TCM) of GBM U-87 cells contains the required developmental factors for tube formation. TCM of treated cells alone and in combination reduced the tube formation in primary endothelial cells. TCM of control cells showed progressive and rich vasculature (Figure 5D).

### PI-103 and EPZ-6438 targets molecular pathways synergistically

**Figure 6:**
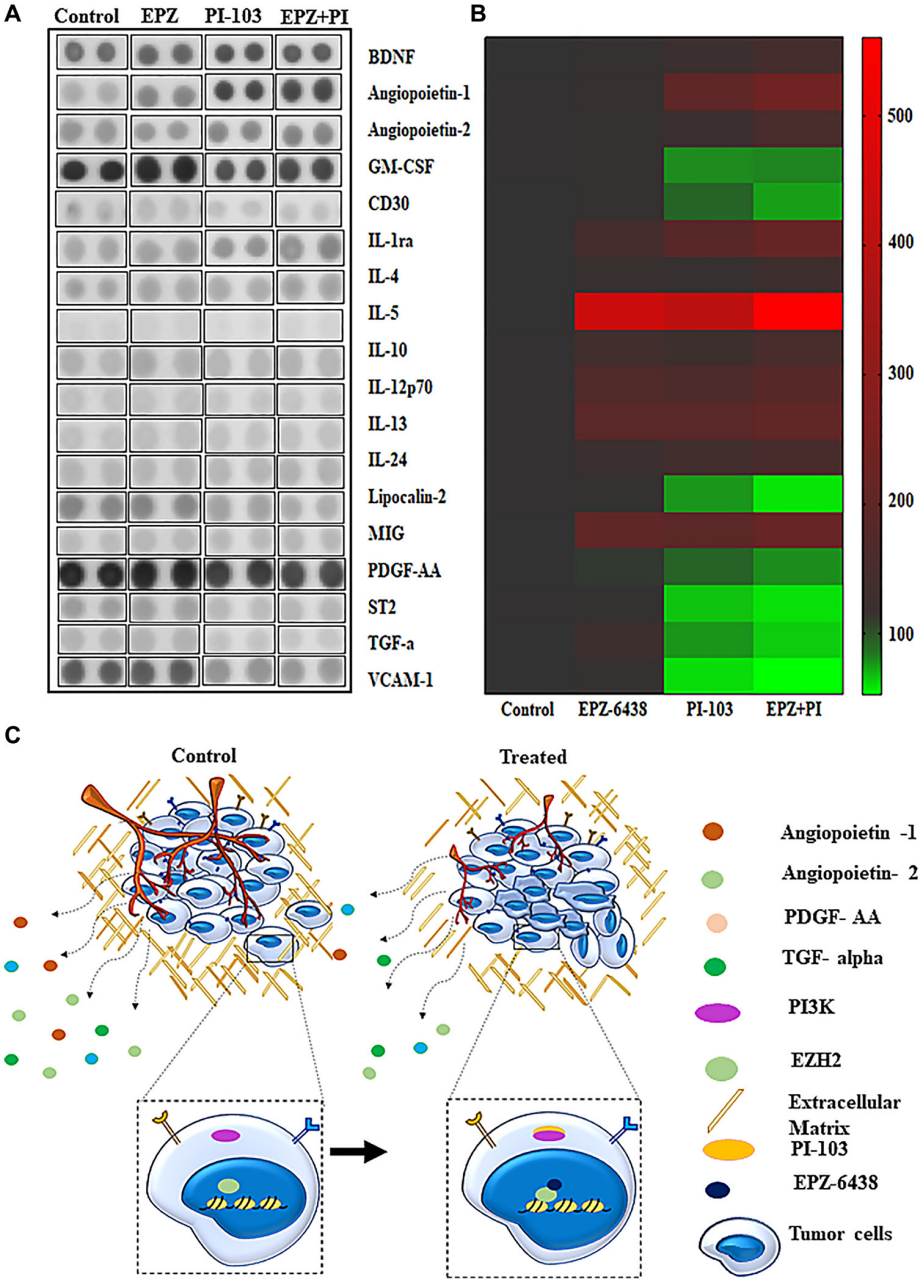
Cytokine analysis explain the key proteins involved in tumor progression. (**A**) Cytokine array analysis showed the various proteins of angiogenesis and metastasis are effectively changed during the course of combination treatment. (**B**) Heat map data depicted the intensity of upregulation and down regulation of all the affected proteins. (**C**) Schematic representation of effect of EPZ-6438 and PI-103 in GBM U-87 cells.

Cytokine proteome profiling data gave the output about the role of cytokines in tumor microenvironment condition. Combination of PI-103 and EPZ-6438 targets multiple proteins and we are listing here few of very impressive outcomes. We have found the downregulation of VCAM-1. Expression of VCAM-1 is finely associated with cancer metastasis in several cancers [38]. In case of glioblastoma, it is associated with clinicopathological conditions. Combination of our inhibitors showed the reduction in VCAM-1 expression and strengthen our data of metastasis inhibition. We did also find downregulation of ST2 (suppression of tumorogenicity-2), which is classified as a intreluikin-1 receptor-like 1. The basic function of ST2 is participation in inflammatory pathways. It is associated with inflammation in tumor microenvironment and progression of several cancers [39, 40]. TNFR superfamily characteristically articulated in hematopoietic malignancies, germ line tumors. CD30 is a member of TNFR superfamily and participated in malignant lymphoma. Its expression is correlated with tumor progression. However, the detailed study of CD30 downregulation in glioblastoma needs to be explored, our data showed downregulation of CD30 in combination treatment [41–43]. We also found upregulated molecules, which are equally important in tumor microenvironment conditions. IL 24 is connected with IL10 subfamily. Many studies explained its crucial participation in tumor suppression. Our results of combination regime showed its upregulation which is also lined with the tumor suppressor properties of IL-24 [44–48]. Another cytokine, IL-10 was also upregulated in our combination treatment. IL-10 is produced by macrophages, T cells and NK cells. IL10 functions as immunosuppressive and antiangiogenic units. It is being utilized for T-cell immune functions and suppression of inflammatory activities associated with cancers [49, 50]. Although we found multiple molecular changes in several proteins’ expression (Figure 6A) however their role in GBM U-87 is not directly connected and need to explored further for their specific roles. Heat map data expresses the ratio of low to high expression of protein molecules with specific targeting (Figure 6B). Schematic representation depicted our observation after the combination regime (Figure 6C).

## DISCUSSION

Glioblastoma multiforme is known as the most common cancer of central nervous system and deadliest form of brain cancer. It includes approximately 52% in total cases of gliomas [51]. GBM (U-87) is associated with diffused necrosis, high proliferation rate of cancer cells and neo-angiogenesis in tumor microenvironment [52, 53]. In our studies we have investigated the role of combination of two different chemical inhibitors on GBM U87 cells. PI3K participates in cancer progression and tumor cells growth and EZH2 worked as a transcriptional repressor in cell growth [54, 55]. Precision Neuro-Oncology resulting from specific and precise targeting of identified gene mutations is a new paradigm for glioblastoma therapy. We started with initial assays to determine specific concentrations of both the molecules. EPZ-6438 did not provide a significant cell death on higher concentrations i.e., 25 μM but PI-103 inhibits the cell viability at 5 μM. We stabilize the PI-103 concentration (5 μM) and vary the EPZ-6438 concentrations to determine the synergistic effect on cancer cell viability and we found that the effective concentration of EPZ-6438 was 5 μM (Supplementary Figure 1).

GBM U-87 cells have the high potential of migration and invasion activities. Glioblastoma migration is a very complex dynamic process, which includes cells interaction with extracellular molecules, cellular structure reorganization for invadopodia and degradation of surrounding matrix through many proteolytic enzymes secreted by tumor cells. Boyden chamber analysis was performed to explore the role of PI-103 and EPZ-6438. Combined regime of PI-103 and EPZ-6438 reduced the cell migration significantly (Figure 1A). Thus, the adhesion properties of GBM (U-87) analyzed along with cytoskeleton reorganization and the data supports the idea of migration inhibition synergistically (Figure 1B). As PI-103 is known for its anti-proliferative effect on cancer cells, we found that cells were stuck in sub GI phase (Figure 2A) during treatment of both the molecules. Synergistic effect increased the population of sub G1. This data gave another hope that cell migration stopped due to cell cycle inhibition. Generally, chronic inflammation increased oxidative stress in brain tissue.

Generation of high amount of reactive oxygen species (ROS) can affect mitochondrial and chromosomal DNA and can lead to damage DNA [56, 57]. This promotes abnormal metabolic activities and genetic instability. Interestingly the basal level of ROS is high in GBM U-87 cells and it participates in regulation in many signaling pathways [58]. We found that ROS expression decreased gradually in treatment with PI-103 and EPZ-6438 (Figure 2B). As mitochondria is one of the main reasons for ROS generation in tumor cells, we found that compromised membrane of mitochondria repair during this treatment (data not shown). This repair mechanism can be the reason of reduced ROS generation and inhibition of cancer progression in GBM U87 cells. Invasive properties were analyzed gradually with Boyden chamber analysis and gelatin degrading assay (Figure 3A and 3B) and we found that MMP 2 degradation is the key reason for inhibiting the invasion property of GBM U-87 cells. Combination of PI-103 and EPZ-6438 showed less expression of MMP2 in zymography analysis (Figure 3C).

The aggression of GBM U-87 is due to reoccurrence after the treatment or therapies in patients. It is reported that a small subset of stem cell population derived the reoccurrence capability for lethality of GBM U87 [8, 30, 59, 60]. Monotherapeutic approaches for continuous Treatments could be a major reason of drug resistance. Small molecule inhibitors can also start such behaviors in tumor cells for long treatment. As we did not find apoptosis, a dose reduction treatment may overcome the effect on resistance in tumor cells. Combination approach with chemo/radiotherapy may also overcome the drug resistance with effective treatment efficacy in patients [61]. GBM known to have a high rate of recurrence due to small subset of stem cells populations i.e., GSCs. GSCs can escape from chemotherapy or radiotherapy and may proliferate after the treatment [26, 62, 63]. Inefficient targeting of these stem cells helps in tumor resistance and recurrence [64–68]. Therapeutic targeting of these stem cells with surface markers, signaling pathways may help to overcome these properties of stem cells. We found a gradual reduction in sphere formation till 12 days and it did not show any sign of recurrence. Our combination approach can be effective along with targeting specific stem cell markers or stem cell specific signaling pathways for the reduction of tumor recurrence. It could be due to participation of PI3k signaling in tumor invasiveness and stemness [69]. Later we observed the metastatic and angiogenic behavior of GBM U-87 cells. We found that combination regime downregulated MENA and A2BR molecule expression (Figure 5A and 5B), which participates in cancer metastasis.

2D angiogenesis showed reduction in tube formation (Figure 5D), which suggested that our molecules significantly target the tumor microenvironment conditions and inhibits GBM U-87 cell progression. To validate further the molecules responsible for metastasis and angiogenesis inhibition, we performed cytokine proteome profiling array analysis. Cytokine profiling data gives a vast information about the molecules and helps in targeting a few interesting molecules which are crucial for angiogenesis and metastasis.

Combination of PI-103 and EPZ-6438 increased the expression of BDNF. Many reports explained that BDNF along with receptor, Tropomyosin receptor kinase B is found to be upregulated in many tumors but recently it has been investigated that BDNF has immuno-augmentation properties and participates in anti-tumor response. Ang2 known to be an inducer of tumor progression and invasion in several cancers [70–72]. However, we found that Ang2 expression is decreased in combination of EPZ-6438 and PI-103. It is widely accepted that MMP2 expression is correlated with the expression of Ang2 in glioblastoma. Selective inhibition of MMP2 at invasive front can hampered the Ang2 induced activities in glioblastoma [73]. In our studies we found the significant inhibition of MMP2 with the combination of EPZ-6438 and PI-103. There may be an activity inhibition of Ang2 occurred in GBM U-87 cells via inhibition of MMP2 and cells are not able to invade and metastasize.

IL-10 is known as the activator of T helper cell, it helps in blocking tissue inflammation and participates in cancerous cell clearance through cellular players. Apart from many interleukins, IL-24 is known as a multifunctional cancer killing interleukins, we found the expression of IL-24 was high in combination of PI-103 and EPZ-6438. Many therapies target VCAM-1 due to its role in adhesion properties. We found that VCAM-1 expression significantly reduced with synergistic effect of our combined regime (Figure 6A). Lipocalin 2 is also named as neutrophil gelatinase-associated lipocalin, has been identified as a potential biomarker in several cancers. Although the function of lipocalin-2 is not well defined but its expression is correlated with progression and metastasis of majority of these cancers [74]. Downregulation of Lipocalin 2 added the new outcomes in glioblastoma. This data shows multiple key molecules (Supplementary Figure 4), which are responsible for tumor progression and angiogenesis.

Our idea of combination regime for GBM U-87 cell treatment gives the insight into many important characteristics of cancerous cells. Glioblastoma multiforme is known to be an aggressive brain tumor and has a high rate of disease recurrence [26, 62, 63]. Increasing resistance in chemotherapy or monotherapy have opened a new window towards combination therapy regime. New innovative combination drugs are being tested in preclinical and clinical trials and many are undergoing in development process. TMZ along with focal chemotherapy is one of the existing typical care of GBM after surgical removal of tumor. Many studies explained about the resistance along with many side effects of TMZ in GBM [8, 75]. Our studies using PI-103 and EPZ-6438 targets PI3K/mTOR and EZH2 respectively. We have shown the potential of inhibition of metastasis and invasion capacities of GBM. Although our data is not showing apoptosis in later stages but may strengthen the combination approach with TMZ and can be a potential way of first line of treatment to GBM. Targeting two or more signaling axes in cancer is more effective than chemotherapy or monotherapy [61, 76] and *in-vitro* and *in-vivo* characterization of such novel molecules led to the development of these drugs to clinical trials in GBM.

We found that significant amount of metastatic and angiogenic proteins are reduced during treatment. Cytokine proteome profiling array data gives a brief of effect of combination treatment and the molecules that need to be further elucidated for better outcomes in novel combination therapies.

## MATERIALS AND METHODS

### Cell line, inhibitors and primary antibodies

GBM (U-87) cell line was obtained from NCCS, Pune. Inhibitor PI-103 was procured from Echelon Biosciences and EPZ-6438 from APExBIO. CD24 antibody, Fluo-4 AM, DCFDA/H2DCFDA and Oregon green 488 were procured from Invitrogen. MENA antibody was purchased from Novus, USA. A2BR antibody was procured from Alomone labs, Israel. Phalloidin- Actin was obtained from Cell signaling. α-tubulin antibody was procured from sigma. β-catenin, SMAD1, SNAI3 were procured from cloud clone (USA). All the media were purchased from HiMEDIA, India.

### Cell cytotoxicity assay

GBM (U-87) cell line were seeded into fresh 24 well plates with 15000 cells/well. After cells were attached for 12–18 hrs, they were treated with PI-103, EPZ-6438 and in combination with different concentration (10 nM, 100 nM, 500 nM, 1 uM, 5 uM, 10 uM and 25 uM) for 48 hrs. After 48 hrs of treatment, cells were harvested using 0.25% trypsin and counted with hemocytometer to get IC50 value of drug as compared to untreated control. After counting we converted cell number into percentage then graph was made as percent reduction of cell number as compared to the drug. The IC50 value of PI-103 is approximately 10 μM, for EPZ-6438 we didn’t get IC50 value and for combination we got less than 5 μM IC50 value. Rest of the experiment were done according to these concentrations only and we used 5 μM of each drug alone and in combination for further experiments.

### Transwell-chamber migration and invasion assay

Transwell inserts (24-well, 8-μm pore size; HiMEDIA) with or without 50 μg/ml Matrigel (BD Biosciences) coating layer were used to assess the cells invasive and migratory abilities, respectively. Pre-treated (48 hrs with PI-103, EPZ-6438 and in combination) GBM U-87 cells were suspended at a final density of 3.5 × 10^4^ cells/mL in a serum-free medium and seeded in the upper well of the chamber. The lower well of the chamber contained media supplemented with 10% FBS. After 24 hrs, cells on the upper surface of the filter were removed using a cotton swab. Cells that had invaded or migrated through the matrigel or filter to the lower surface were fixed with 4% paraformaldehyde and stained with 1% crystal violet. Cells were counted from 3 randomly selected fields per each chamber.

### Wound healing assay

Wound healing assay was performed in a 12 well culture plate. Cells were grown in 12-well tissue culture plate. Cells were grown up to 80 to 90% confluency. A scratch was made by a 10 μL pipette micro tip through the center of all the wells to create an artificial and uniform wound. Culture media removed and fresh media with and without drug were added. Then migratory properties analyzed by taking picture under microscope (10X DIC) at 0 hr, 12 hrs and 24 hrs. Wound healing rate was calculated by counting five to seven fields per image. Migration rate calculated and converted into percentage wound closer in reference to control.

### Immunofluorescence assay

The assay was performed as described in our previous paper [77]. Briefly, GBM U-87 cells were seeded on coverslip and treated with/without drug (PI-103, EPZ-6438 and in combination). Cells were probed with phalloidin β-Actin (Cell signaling) and α-Tubulin (Sigma) and image were taken under microscope (60X Nikon fluorescent microscope).

### Cell adhesion assay

Cells were seeded in 60 mm dish and treated with drugs as mentioned above for 48 hr. Cells were harvested and re-suspended in media. Meanwhile 12 well plate coated with 50 μM Poly-L-lysine for 1 hr at RT. Treated/untreated GBM U-87 cells were harvested and suspended at a final density of 3 × 10^5^ cells/ml and plated on Poly-L-lysine coated plates for 30 mins. Thereafter unattached cells were removed by inverting the 12 well plate and gently washed with 1X PBS twice. Attached cells were fixed with ice-cold methanol for 10 mins at RT. Washed with 1X PBS and stained with 1% crystal violet stain for 5 mins with gentle shaking. Plate was washed with water and kept for drying. After that image was taken by color camera and stain solubilized into 1 ml of 1% SDS for 1 hr and collected in fresh 1.5 ml centrifuge tubes. Absorbance was taken at 595 nm (Bio-Rad microplate reader) in 96 well plate in 4 replicates. Absorbance value was converted into percentage and graph was made accordingly.

### Cell cycle analysis

GBM U-87 cells were treated with drugs as mentioned above for 48 hrs. Meanwhile hypotonic solution (100 ml DDW, 100 mg Na-citrate, 4 mg RNase A, 5 mg Propidium Iodide, 30 μl Tween20) was prepared with or without PI stain. Cells were washed with 1XPBS. Hypotonic solution was added to the monolayer of cells and collected using cell scraper. Cells were shaken vigorously and dislodged by fine tipped pipette. Treated/untreated cells were transferred to 1.5 ml centrifuge tube and centrifuged at 100 RCF and the nuclear pellet was suspended in fresh hypotonic PI solution. Sample was run in FACS canto II on the same day.

### DCFDA/ROS analysis

GBM U-87 cells were treated for 48 hrs as mentioned above. After 48 hrs of treatment with PI-103, EPZ-6438 and in combination cells were washed with 1XPBS and 5 μM of DCFDA (Invitrogen) was added for 30 mins in complete media. After that cells were trypsinized and collected in 1.5 ml centrifuge tube. Cells were washed with 1X PBS in to 0.1% FBS and re-suspended the cells in PBS/FBS solution. Flow Cytometry analysis was done on same day by BD FACS CANTO II.

### Invadopodia assay

GBM U-87 cells were harvested and seeded into 60 mm dish for invadopodia experiment. The cells were treated with specific drugs for 48 hrs as mentioned above. Prepared fluorescent gelatin coated coverslips according to the protocol [78]. Briefly, U87 cells were harvested and plated on coverslip in 60 to 70% confluency which was already coated with Gelatin labeled with Oregon green 488. After that coverslip with the cells was transferred to the CO_2_ incubator for 14–16 hours for initiation of the gelatin degradation. After 14–16 hrs of incubation the coverslip was taken out from the plate and quickly fixed with 4% formaldehyde for 10–15 min at room temperature. Fixation solution was removed and washed with 1X PBS twice, blocking and permeabilization was performed with solution (3% BSA in 1X PBS containing 0.1% Triton X-100) for 15–20 min at room temperature in dark. Blocking solution was removed and washed with 1X PBS twice. This was probed with Alexa Fluor 555 Actin-Phalloidin (1:500 dilution) for 30–40 min in dark. Phalloidin was removed and washed with 1X PBS twice and thereafter the coverslip was mounted by inverting over a glass slide containing a drop of mounting medium ProLong™ Gold antifade reagent with DAPI. Slides were dried and sealed with colorless Nail paint. Images were taken under Nikon Fluorescent Microscope (at 60X) and quantified [80].

### CD24 marker staining

GBM U-87 cells were seeded in 60 mm dish and treated with drugs as mentioned above for 48 hrs. After 48 hours of treatment, cells were harvested using trypsin. Cells were washed with 1X PBS 1 to 2 times and then re-suspended in 1X PBS+0.1% FBS solution. Human CD24 FITC (Invitrogen, Fluorescein isothiocyanate) antibody was conjugated at a concentration of 1:100 for 45 minutes in dark at RT. Cells were centrifuged and pelleted down after 45 minutes and resuspended in 500 μl of PBS/FBS solution. The cells were analyzed using BD FACS CANTO II on the same day.

### Western blot analysis

Effect of treatment of PI-103 and EPZ-6438 on metastatic proteins was analyzed as described in previous studies [79]. Cells were treated and harvested after 48 hrs. Protein lysates were ran on to SDS PAGE for 3 hrs. The gel was transferred onto the nitrocellulose membrane and incubated with antibodies O/N at 4°C. Membrane was developed and analyzed.

### Sphere formation assay

Growth media preparation: Incomplete media (MEM) 24 ml, Hydrocortisone (10 mg/ml) 1 ml, Insulin (10 mg/ml) 100 μl, EGF (100 μg/ml) 5 μl and 500 μl of B-27 (50X) were mixed gently and kept in 4°C for use in sphere formation. Twelve well plate coated with 1% low melting agarose (HiMEDIA) in incomplete MEM media. GBM (U-87) (U87) Cells were harvested and seeded in low melting agarose and plates were coated with 2500 cells/well in previously prepared growth media with and without drug. Every 3rd day growth media was added with drug up to 12 days. After 12 days spheres were counted and images were taken under the light microscope (20X). Treated/untreated sphere were collected and centrifuged at 2500 rpm for 5 mins. After that sphere cells dislodged using accutase and sphere cells were also counted and graph was made as percent reduction of spheres and sphere cells.

### Gelatin zymography assay

Treated/untreated tissue culture conditioned (CM) media were collected and concentrated using concentrator. Equal amount of protein containing CM was loaded on 10% SDS gel containing 2% gelatin. Then gel was run on 60V for 2–3 hrs. After that washed the gel with renaturation buffer (2.5% Triton X-100) 3 times for 20 mis each. Gel was kept in developing buffer (premade HiMEDIA) for 16–18 hrs and stained with Coomassie brilliant blue stain for 1 hr. MMP-2 band was detected after destaining the gel. Picture was taken using a color camera.

### Tube formation assay and cytokine array

Tube formation assay and cytokine array analysis were conducted as described previously [80]. Briefly, primary endothelial cells were cultured and incubated with conditioned media of treated and untreated cells. Tube formation was analyzed after 4 hrs and images were taken and analyzed by AngioTool software. For cytokine proteome array analysis, CM of all treated cells was collected and quantified. Equal amount of protein was loaded on each membrane for 24 hrs and developed. The data was quantified with ImageJ.

## SUPPLEMENTARY MATERIALS



## References

[1] WHO. GLOBOCAN 2018-HOME. 2018 https://gco.iarc.fr/.

[2] Stupp R , Mason WP , van den Bent MJ , Weller M , Fisher B , Taphoorn MJ , Belanger K , Brandes AA , Marosi C , Bogdahn U , Curschmann J , Janzer RC , Ludwin SK , et al , and European Organisation for Research and Treatment of Cancer Brain Tumor and Radiotherapy Groups, and National Cancer Institute of Canada Clinical Trials Group. Radiotherapy plus concomitant and adjuvant temozolomide for glioblastoma. N Engl J Med. 2005; 352:987–96. 10.1056/nejmoa043330. 15758009

[3] Chen J , Li Y , Yu TS , McKay RM , Burns DK , Kernie SG , Parada LF . A restricted cell population propagates glioblastoma growth after chemotherapy. Nature. 2012; 488:522–6. 10.1038/nature11287. 22854781 PMC3427400

[4] Wen PY , Kesari S . Malignant gliomas in adults. N Engl J Med. 2008; 359:492–507. 10.1056/nejmra0708126. 18669428

[5] Jain RK , Di Tomaso E , Duda DG , Loeffler JS , Sorensen AG , Batchelor TT . Angiogenesis in brain tumours. Nat Rev Neurosci. 2007; 8:610–22. 10.1038/nrn2175. 17643088

[6] Vredenburgh JJ , Desjardins A , Herndon JE 2nd , Dowell JM , Reardon DA , Quinn JA , Rich JN , Sathornsumetee S , Gururangan S , Wagner M , Bigner DD , Friedman AH , Friedman HS . Phase II trial of bevacizumab and irinotecan in recurrent malignant glioma. Clin Cancer Res. 2007; 13:1253–9. 10.1158/1078-0432.ccr-06-2309. 17317837

[7] Filatova A , Acker T , Garvalov BK . The cancer stem cell niche (s): the crosstalk between glioma stem cells and their microenvironment. Biochim Biophys Acta. 2013; 1830:2496–508. 10.1016/j.bbagen.2012.10.008. 23079585

[8] Bao S , Wu Q , McLendon RE , Hao Y , Shi Q , Hjelmeland AB , Dewhirst MW , Bigner DD , Rich JN . Glioma stem cells promote radioresistance by preferential activation of the DNA damage response. Nature. 2006; 444:756–60. 10.1038/nature05236. 17051156

[9] Eramo A , Ricci-Vitiani L , Zeuner A , Pallini R , Lotti F , Sette G , Pilozzi E , Larocca LM , Peschle C , De Maria R . Chemotherapy resistance of glioblastoma stem cells. Cell Death Differ. 2006; 13:1238–41. 10.1038/sj.cdd.4401872. 16456578

[10] Taniuchi K , Nishimori I , Hollingsworth MA . Intracellular CD24 inhibits cell invasion by posttranscriptional regulation of BART through interaction with G3BP. Cancer Res. 2011; 71:895–905. 10.1158/0008-5472.can-10-2743. 21266361

[11] Deng J , Gao G , Wang L , Wang T , Yu J , Zhao Z . CD24 expression as a marker for predicting clinical outcome in human gliomas. BioMed Research International. 2012; 2012:517172. 10.1155/2012/517172. 22500096 PMC3303885

[12] Raza M , Prasad P , Gupta P , Kumar N , Sharma T , Rana M , Goldman A , Sehrawat S . Perspectives on the role of brain cellular players in cancer-associated brain metastasis: translational approach to understand molecular mechanism of tumor progression. Cancer Metastasis Rev. 2018; 37:791–804. 10.1007/s10555-018-9766-5. 30284650

[13] Salazar-Ramiro A , Ramírez-Ortega D , Pérez de la Cruz V , Hérnandez-Pedro NY , González-Esquivel DF , Sotelo J , Pineda B . Role of redox status in development of glioblastoma. Front Immunol. 2016; 7:156. 10.3389/fimmu.2016.00156. 27199982 PMC4844613

[14] Weathers SP , Gilbert MR . Advances in treating glioblastoma. F1000Prime Rep. 2014; 6:46. 10.12703/p6-46. 24991423 PMC4047946

[15] Hadjipanayis CG , Van Meir EG . Tumor initiating cells in malignant gliomas: biology and implications for therapy. J Mol Med (Berl). 2009; 87:363–74. 10.1007/s00109-009-0440-9. 19189072 PMC2693383

[16] Zhao HF , Wang J , Shao W , Wu CP , Chen ZP , To SST , Li WP . Recent advances in the use of PI3K inhibitors for glioblastoma multiforme: current preclinical and clinical development. Mol Cancer. 2017; 16:100. 10.1186/s12943-017-0670-3. 28592260 PMC5463420

[17] Wen PY , Lee EQ , Reardon DA , Ligon KL , Alfred Yung W . Current clinical development of PI3K pathway inhibitors in glioblastoma. Neuro-oncol. 2012; 14:819–29. 10.1093/neuonc/nos117. 22619466 PMC3379803

[18] Viré E , Brenner C , Deplus R , Blanchon L , Fraga M , Didelot C , Morey L , Van Eynde A , Bernard D , Vanderwinden JM , Bollen M , Esteller M , Di Croce L , et al. The Polycomb group protein EZH2 directly controls DNA methylation. Nature. 2006; 439:871–4. 10.1038/nature04431. 16357870

[19] Yoo KH , Hennighausen L . EZH2 methyltransferase and H3K27 methylation in breast cancer. Int J Biol Sci. 2012; 8:59–65. 10.7150/ijbs.8.59. 22211105 PMC3226033

[20] Zingg D , Debbache J , Schaefer SM , Tuncer E , Frommel SC , Cheng P , Arenas-Ramirez N , Haeusel J , Zhang Y , Bonalli M , McCabe MT , Creasy CL , Levesque MP , et al. The epigenetic modifier EZH2 controls melanoma growth and metastasis through silencing of distinct tumour suppressors. Nat Commun. 2015; 6:6051. 10.1038/ncomms7051. 25609585

[21] Konze KD , Ma A , Li F , Barsyte-Lovejoy D , Parton T , Macnevin CJ , Liu F , Gao C , Huang XP , Kuznetsova E , Rougie M , Jiang A , Pattenden SG , et al. An orally bioavailable chemical probe of the lysine methyltransferases EZH2 and EZH1. ACS Chem Biol. 2013; 8:1324–34. 10.1021/cb400133j. 23614352 PMC3773059

[22] Yang D , Hou T , Li L , Chu Y , Zhou F , Xu Y , Hou X , Song H , Zhu K , Hou Z , Peng H , Jia H . Smad1 promotes colorectal cancer cell migration through Ajuba transactivation. Oncotarget. 2017; 8:110415–25. 10.18632/oncotarget.22780. 29299158 PMC5746393

[23] Larsen BD , Megeney LA . Parole terms for a killer: Directing the caspase3/CAD induced DNA strand breaks to coordinate changes in gene expression. Cell Cycle. 2010; 9:2940–45. 10.4161/cc.9.15.12335. 20714221 PMC3040922

[24] Ritch SJ , Brandhagen BN , Goyeneche AA , Telleria CM . Advanced assessment of migration and invasion of cancer cells in response to mifepristone therapy using double fluorescence cytochemical labeling. BMC Cancer. 2019; 19:376. 10.1186/s12885-019-5587-3. 31014286 PMC6480622

[25] Quintero-Fabián S , Arreola R , Becerril-Villanueva E , Torres-Romero JC , Arana-Argáez VE , Lara-Riegos J , Ramírez-Camacho MA , Alvarez Sanchez ME . Role of matrix metalloproteinases in angiogenesis and cancer. Front Oncol. 2019; 9:1370. 10.3389/fonc.2019.01370. 31921634 PMC6915110

[26] Sheikhpour E , Noorbakhsh P , Foroughi E , Farahnak S , Nasiri R , Neamatzadeh H . A survey on the role of interleukin-10 in breast cancer: A narrative. Rep Biochem Mol Biol. 2018; 7:30–37. 30324115 PMC6175593

[27] Valkenburg KC , Graveel CR , Zylstra-Diegel CR , Zhong Z , Williams BO . Wnt/β-catenin Signaling in Normal and Cancer Stem Cells. Cancers (Basel). 2011; 3:2050–79. 10.3390/cancers3022050. 24212796 PMC3757404

[28] Wang Y , Shi J , Chai K , Ying X , Zhou BP . The role of snail in EMT and tumorigenesis. Curr Cancer Drug Targets. 2013; 13:963–72. 10.2174/15680096113136660102. 24168186 PMC4004763

[29] Barrallo-Gimeno A , Nieto MA . The Snail genes as inducers of cell movement and survival: implications in development and cancer. Development. 2005; 132:3151–61. 10.1242/dev.01907. 15983400

[30] Venere M , Fine HA , Dirks PB , Rich JN . Cancer stem cells in gliomas: identifying and understanding the apex cell in cancer's hierarchy. Glia. 2011; 59:1148–54. 10.1002/glia.21185. 21547954 PMC3107874

[31] Cuddapah VA , Robel S , Watkins S , Sontheimer H . A neurocentric perspective on glioma invasion. Nat Rev Neurosci. 2014; 15:455–65. 10.1038/nrn3765. 24946761 PMC5304245

[32] Lamszus K , Kunkel P , Westphal M . Invasion as limitation to anti-angiogenic glioma therapy. Acta Neurochir Suppl. 2003; 88:169–77. 10.1007/978-3-7091-6090-9_23. 14531575

[33] Shah A , Redhu R , Nadkarni T , Goel A . Supratentorial glioblastoma multiforme with spinal metastases. J Craniovertebr Junction Spine. 2010; 1:126–29. 10.4103/0974-8237.77678. 21572635 PMC3075830

[34] Vertosick FT Jr , Selker RG . Brain stem and spinal metastases of supratentorial glioblastoma multiforme: a clinical series. Neurosurgery. 1990; 27:516–22. 10.1097/00006123-199010000-00002. 2172859

[35] Oudin MJ , Barbier L , Schäfer C , Kosciuk T , Miller MA , Han S , Jonas O , Lauffenburger DA , Gertler FB . MENA confers resistance to paclitaxel in triple-negative breast cancer. Mol Cancer Ther. 2017; 16:143–55. 10.1158/1535-7163.mct-16-0413. 27811011 PMC5359014

[36] Monteith GR , Prevarskaya N , Roberts-Thomson SJ . The calcium–cancer signalling nexus. Nat Rev Cancer. 2017; 17:367–80. 10.1038/nrc.2017.18. 28386091

[37] Monteith GR , Davis FM , Roberts-Thomson SJ . Calcium channels and pumps in cancer: changes and consequences. J Biol Chem. 2012; 287:31666–73. 10.1074/jbc.r112.343061. 22822055 PMC3442501

[38] Hu B , Guo P , Fang Q , Tao HQ , Wang D , Nagane M , Huang HJ , Gunji Y , Nishikawa R , Alitalo K , Cavenee WK , Cheng SY . Angiopoietin-2 induces human glioma invasion through the activation of matrix metalloprotease-2. Proc Natl Acad Sci U S A. 2003; 100:8904–9. 10.1073/pnas.1533394100. 12861074 PMC166411

[39] Kong DH , Kim YK , Kim MR , Jang JH , Lee S . Emerging roles of vascular cell adhesion molecule-1 (VCAM-1) in immunological disorders and cancer. Int J Mol Sci. 2018; 19:1057. 10.3390/ijms19041057. 29614819 PMC5979609

[40] Hong J , Kim S , Lin PC . Interleukin-33 and ST2 signaling in tumor microenvironment. J Interferon Cytokine Res. 2019; 39:61–71. 10.1089/jir.2018.0044. 30256696 PMC6350413

[41] Chang CP , Hu MH , Hsiao YP , Wang YC . ST2 Signaling in the Tumor Microenvironment. Adv Exp Med Biol. 2020; 1240:83–93. 10.1007/978-3-030-38315-2_7. 32060890

[42] Dürkop H , Latza U , Hummel M , Eitelbach F , Seed B , Stein H . Molecular cloning and expression of a new member of the nerve growth factor receptor family that is characteristic for Hodgkin's disease. Cell. 1992; 68:421–7. 10.1016/0092-8674(92)90180-k. 1310894

[43] Smith CA , Gruss HJ , Davis T , Anderson D , Farrah T , Baker E , Sutherland GR , Brannan CI , Copeland NG , Jenkins NA , Grabstein KH , Gliniak B , McAlister IB , et al. CD30 antigen, a marker for Hodgkin's lymphoma, is a receptor whose ligand defines an emerging family of cytokines with homology to TNF. Cell. 1993; 73:1349–60. 10.1016/0092-8674(93)90361-s. 8391931

[44] van der Weyden CA , Pileri SA , Feldman AL , Whisstock J , Prince HM . Understanding CD30 biology and therapeutic targeting: a historical perspective providing insight into future directions. Blood Cancer J. 2017; 7:e603. 10.1038/bcj.2017.85. 28885612 PMC5709754

[45] Jia Y , Ji K , Ji J , Hao C , Ye L , Sanders AJ , Jiang WG . IL24 and its Receptors Regulate Growth and Migration of Pancreatic Cancer Cells and Are Potential Biomarkers for IL24 Molecular Therapy. Anticancer Res. 2016; 36:1153–63. 26977011

[46] Chada S , Mhashilkar AM , Ramesh R , Mumm JB , Sutton RB , Bocangel D , Zheng M , Grimm EA , Ekmekcioglu S . Bystander activity of Ad-mda7: human MDA-7 protein kills melanoma cells via an IL-20 receptor-dependent but STAT3-independent mechanism. Mol Ther. 2004; 10:1085–95. 10.1016/j.ymthe.2004.08.020. 15564140

[47] Su ZZ , Lebedeva IV , Gopalkrishnan RV , Goldstein NI , Stein C , Reed JC , Dent P , Fisher PB . A combinatorial approach for selectively inducing programmed cell death in human pancreatic cancer cells. Proc Natl Acad Sci U S A. 2001; 98:10332–7. 10.1073/pnas.171315198. 11526239 PMC56961

[48] Su ZZ , Lebedeva IV , Sarkar D , Gopalkrishnan RV , Sauane M , Sigmon C , Yacoub A , Valerie K , Dent P , Fisher PB . Melanoma differentiation associated gene-7, mda-7/IL-24, selectively induces growth suppression, apoptosis and radiosensitization in malignant gliomas in a p53-independent manner. Oncogene. 2003; 22:1164–80. 10.1038/sj.onc.1206062. 12606943

[49] Yacoub A , Mitchell C , Lister A , Lebedeva IV , Sarkar D , Su ZZ , Sigmon C , McKinstry R , Ramakrishnan V , Qiao L , Broaddus WC , Gopalkrishnan RV , Grant S , et al. Melanoma differentiation-associated 7 (interleukin 24) inhibits growth and enhances radiosensitivity of glioma cells *in vitro* and *in vivo* . Clin Cancer Res. 2003; 9:3272–81. 12960112

[50] Dennis KL , Blatner NR , Gounari F , Khazaie K . Current status of IL-10 and regulatory T-cells in cancer. Curr Opin Oncol. 2013; 25:637–45. 10.1097/cco.0000000000000006. 24076584 PMC4322764

[51] AANS. Glioblastoma Multiforme. American Association of Neurological Surgeons. https://www.aans.org/en/Patients/Neurosurgical-Conditions-and-Treatments/Glioblastoma-Multiforme.

[52] Lopez-Gonzalez MA , Sotelo J . Brain tumors in Mexico: characteristics and prognosis of glioblastoma. Surg Neurol Int. 2000; 53:157–62. 10.1016/s0090-3019(99)00177-9. 10713194

[53] Wick W , Platten M , Weller M . New (alternative) temozolomide regimens for the treatment of glioma. Neuro-oncol. 2009; 11:69–79. 10.1215/15228517-2008-078. 18772354 PMC2718961

[54] Thorpe LM , Yuzugullu H , Zhao JJ . PI3K in cancer: divergent roles of isoforms, modes of activation and therapeutic targeting. Nat Rev Cancer. 2015; 15:7–24. 10.1038/nrc3860. 25533673 PMC4384662

[55] Kim KH , Roberts CW . Targeting EZH2 in cancer. Nat Med. 2016; 22:128–34. 10.1038/nm.4036. 26845405 PMC4918227

[56] Jackson SP , Bartek J . The DNA-damage response in human biology and disease. Nature. 2009; 461:1071–8. 10.1038/nature08467. 19847258 PMC2906700

[57] Lahmar Q , Keirsse J , Laoui D , Movahedi K , Van Overmeire E , Van Ginderachter JA . Tissue-resident versus monocyte-derived macrophages in the tumor microenvironment. Biochim Biophys Acta. 2016; 1865:23–34. 10.1016/j.bbcan.2015.06.009. 26145884

[58] Altieri F , Grillo C , Maceroni M , Chichiarelli S . DNA damage and repair: from molecular mechanisms to health implications. Antioxid Redox Signal. 2008; 10:891–938. 10.1089/ars.2007.1830. 18205545

[59] Sadahiro H , Yoshikawa K , Ideguchi M , Kajiwara K , Ishii A , Ikeda E , Owada Y , Yasumoto Y , Suzuki M . Pathological features of highly invasive glioma stem cells in a mouse xenograft model. Brain Tumor Pathol. 2014; 31:77–84. 10.1007/s10014-013-0149-x. 23670138

[60] Cheng L , Wu Q , Guryanova OA , Huang Z , Huang Q , Rich JN , Bao S . Elevated invasive potential of glioblastoma stem cells. Biochem Biophys Res Commun. 2011; 406:643–8. 10.1016/j.bbrc.2011.02.123. 21371437 PMC3065536

[61] Choi JS , Kim CS , Berdis A . Inhibition of translesion DNA synthesis as a novel therapeutic strategy to treat brain cancer. Cancer Res. 2018; 78:1083–96. 10.1158/0008-5472.can-17-2464. 29259011

[62] Singh SK , Hawkins C , Clarke ID , Squire JA , Bayani J , Hide T , Henkelman RM , Cusimano MD , Dirks PB . Identification of human brain tumour initiating cells. Nature. 2004; 432:396–401. 10.1038/nature03128. 15549107

[63] Liu G , Yuan X , Zeng Z , Tunici P , Ng H , Abdulkadir IR , Lu L , Irvin D , Black KL , Yu JS . Analysis of gene expression and chemoresistance of CD133+ cancer stem cells in glioblastoma. Mol Cancer. 2006; 5:67. 10.1186/1476-4598-5-67. 17140455 PMC1697823

[64] Ghosh D , Nandi S , Bhattacharjee S . Combination therapy to checkmate Glioblastoma: clinical challenges and advances. Clin Transl Med. 2018; 7:33. 10.1186/s40169-018-0211-8. 30327965 PMC6191404

[65] Anjum K , Shagufta BI , Abbas SQ , Patel S , Khan I , Shah SA , Akhter N , Hassan SS . Current status and future therapeutic perspectives of glioblastoma multiforme (GBM) therapy: A review. Biomed Pharmacother. 2017; 92:681–89. 10.1016/j.biopha.2017.05.125. 28582760

[66] Arcella A , Palchetti S , Digiacomo L , Pozzi D , Capriotti AL , Frati L , Oliva MA , Tsaouli G , Rota R , Screpanti I , Mahmoudi M , Caracciolo G . Brain targeting by liposome–biomolecular corona boosts anticancer efficacy of temozolomide in glioblastoma cells. ACS Chem Neurosci. 2018; 9:3166–74. 10.1021/acschemneuro.8b00339. 30015470

[67] Chowdhury FA , Hossain MK , Mostofa A , Akbor MM , Bin Sayeed MS . Therapeutic potential of thymoquinone in glioblastoma treatment: targeting major gliomagenesis signaling pathways. BioMed Res Int. 2018; 2018:4010629. 10.1155/2018/4010629. 29651429 PMC5831880

[68] Jiang Y , Wang X , Zhang J , Lai R . MicroRNA-599 suppresses glioma progression by targeting RAB27B. Oncol Lett. 2018; 16:1243–52. 10.3892/ol.2018.8727. 29963197 PMC6019909

[69] Han H , Bourboulia D , Jensen-Taubman S , Isaac B , Wei B , Stetler-Stevenson W . An endogenous inhibitor of angiogenesis inversely correlates with side population phenotype and function in human lung cancer cells. Oncogene. 2014; 33:1198–206. 10.1038/onc.2013.61. 23474755 PMC6322540

[70] Yu CF , Chen FH , Lu MH , Hong JH , Chiang CS . Dual roles of tumour cells-derived matrix metalloproteinase 2 on brain tumour growth and invasion. Br J Cancer. 2017; 117:1828–36. 10.1038/bjc.2017.362. 29065106 PMC5729475

[71] Ahmad SA , Liu W , Jung YD , Fan F , Reinmuth N , Bucana CD , Ellis LM . Differential expression of angiopoietin-1 and angiopoietin-2 in colon carcinoma. A possible mechanism for the initiation of angiogenesis. Cancer. 2001; 92:1138–43. 10.1002/1097-0142(20010901)92:5<1138::aid-cncr1431>3.0.co;2-l. 11571726

[72] Ahmad SA , Liu W , Jung YD , Fan F , Wilson M , Reinmuth N , Shaheen RM , Bucana CD , Ellis LM . The effects of angiopoietin-1 and -2 on tumor growth and angiogenesis in human colon cancer. Cancer Res. 2001; 61:1255–9. 11245414

[73] Sfiligoi C , de Luca A , Cascone I , Sorbello V , Fuso L , Ponzone R , Biglia N , Audero E , Arisio R , Bussolino F , Sismondi P , De Bortoli M . Angiopoietin-2 expression in breast cancer correlates with lymph node invasion and short survival. Int J Cancer. 2003; 103:466–74. 10.1002/ijc.10851. 12478661

[74] Zhang Q , Xiang W , Yi DY , Xue BZ , Wen WW , Abdelmaksoud A , Xiong NX , Jiang XB , Zhao HY , Fu P . Current status and potential challenges of mesenchymal stem cell-based therapy for malignant gliomas. Stem Cell Res Ther. 2018; 9:228. 10.1186/s13287-018-0977-z. 30143053 PMC6109313

[75] Trinh VA , Patel SP , Hwu WJ . The safety of temozolomide in the treatment of malignancies. Expert Opin Drug Saf. 2009; 8:493–9. 10.1517/14740330902918281. 19435405

[76] Bayat Mokhtari R , Homayouni TS , Baluch N , Morgatskaya E , Kumar S , Das B , Yeger H . Combination therapy in combating cancer. Oncotarget. 2017; 8:38022–43. 10.18632/oncotarget.16723. 28410237 PMC5514969

[77] Kumar N , Gupta S , Dabral S , Singh S , Sehrawat S . Role of exchange protein directly activated by cAMP (EPAC1) in breast cancer cell migration and apoptosis. Mol Cell Biochem. 2017; 430:115–25. 10.1007/s11010-017-2959-3. 28210903

[78] Díaz B . Invadopodia detection and gelatin degradation assay. Bio Protoc. 2013; 3:e997. 10.21769/bioprotoc.997. 30443559 PMC6233998

[79] Yadav N , Kumar N , Prasad P , Shirbhate S , Sehrawat S , Lochab B . Stable dispersions of covalently tethered polymer improved graphene oxide nanoconjugates as an effective vector for siRNA delivery. ACS Appl Mater Interfaces. 2018; 10:14577–93. 10.1021/acsami.8b03477. 29634909

[80] Kumar N , Prasad P , Jash E , Jayasundar S , Singh I , Alam N , Murmu N , Somashekhar S , Goldman A , Sehrawat S . cAMP regulated EPAC1 supports microvascular density, angiogenic and metastatic properties in a model of triple negative breast cancer. Carcinogenesis. 2018; 39:1245–53. 10.1093/carcin/bgy090. 29982410 PMC6454463

