# Supplementary material for: Amalgamation of PI3K and EZH2 blockade synergistically regulates invasion and angiogenesis: combination therapy for glioblastoma multiforme
Source: Oncotarget. 2020 Dec 22;11(51):4754–69. doi: 10.18632/oncotarget.27842 (PMC7771717; doi:10.18632/oncotarget.27842)
Supplement: Supplementary file 1 [file oncotarget-11-4754-s001.pdf]

Amalgamation of PI3K and EZH2 blockade synergistically regulates invasion and angiogenesis: combination therapy for glioblastoma multiforme

SUPPLEMENTARY MATERIALS

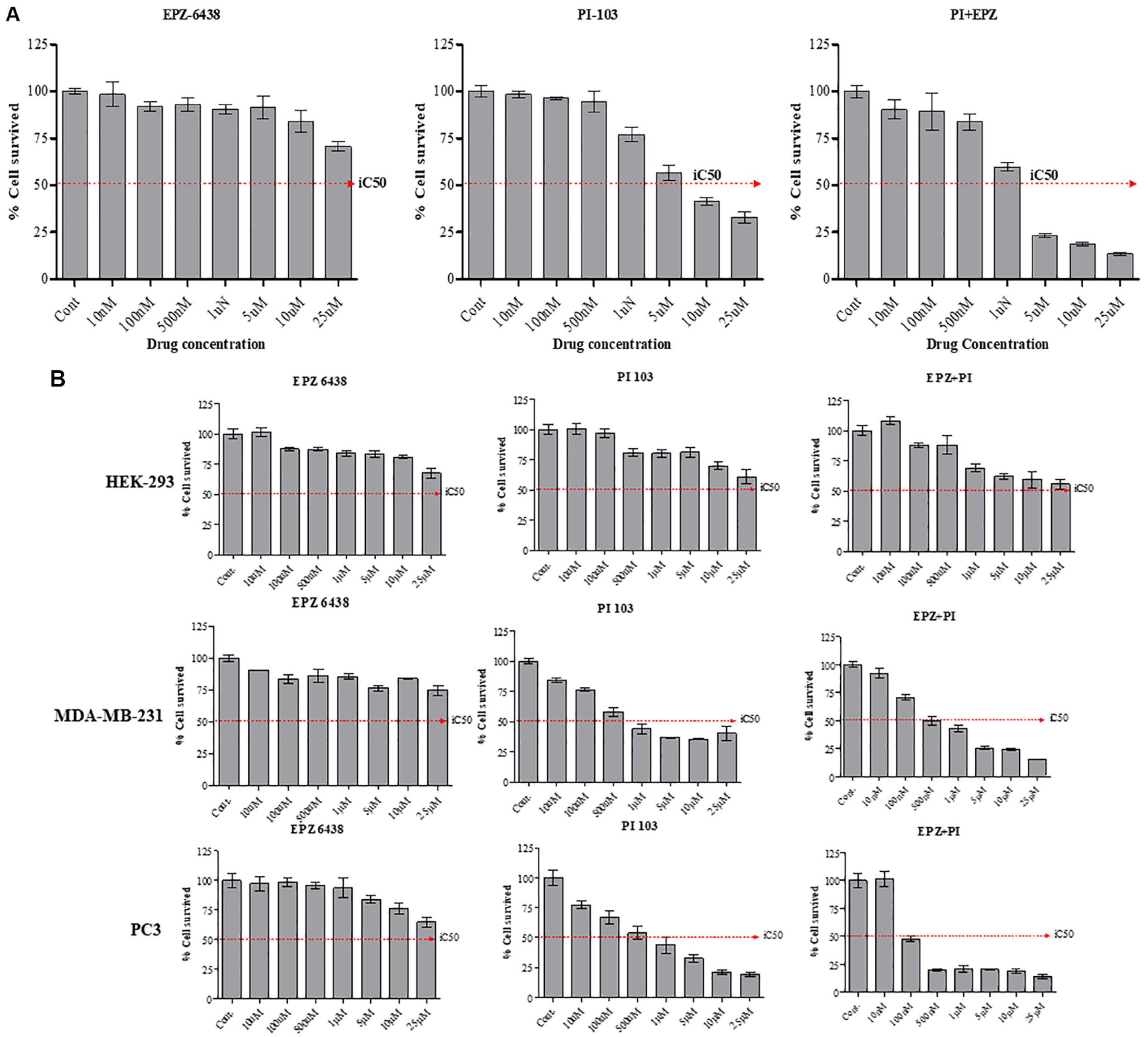

Supplementary Figure 1: Cytotoxicity analysis (cell counting assay). (**A**) PI-103 & EPZ-6438 cell cytotoxicity was calculated in GBM (U87-MG) (U87) cells. 15K cells/well plated in 24 well plate and kept for 24 hrs in CO2 incubator. Cells were treated with drug individually in different concentration for 48 hrs. After 48 hrs cells were counted separately and numerical value were calculated in percent cell death. In case of PI-103 iC50 value was around 10 uM but in the case of EPZ-6438 iC50 value was not found up to 25 uM concentration. Percent cell death increase in combination treated wells (iC50 less than 5 uM). (**B**) PI-103 & EPZ-6438 cell cytotoxicity was calculated in HEK-293, MDA-MB-231 cells and PC3 cells. Based on our data comparison with HEK cells we could find the effectiveness of our combinations on other two cancerous cells lines. Our combination treatment was very effective on PC3 cells. Combination regime is working at very low concentrations comparative to GBM U-87 cells.

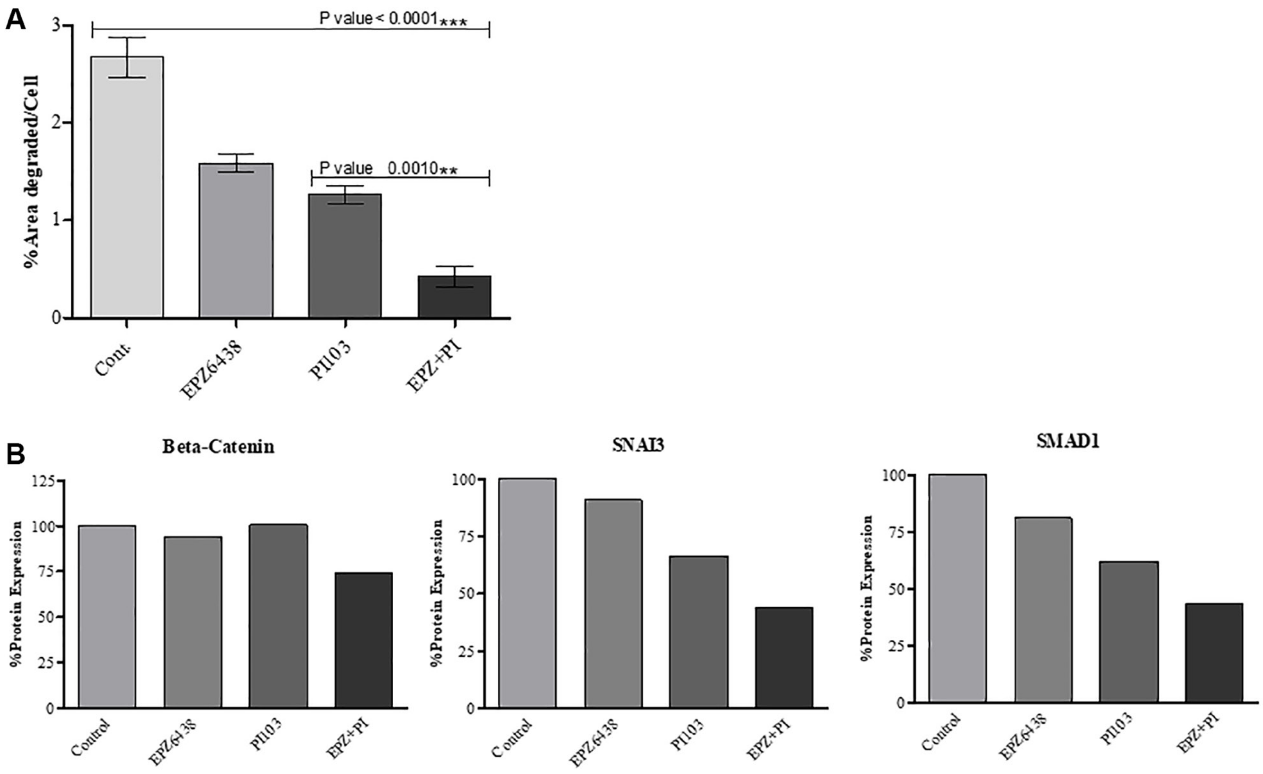

Supplementary Figure 2: Combination of EPZ-6438 and PI-103 targets metastasis in GBM (U87-MG) (U87) cells. (**A**) Quantification of degrading area of invading cells were calculated by Image J. Data quantified in triplicate and showed the effect of combination on cellular invasion capabilities. (**B**) Western blot analysis conducted and showed the reduction of expression of β-catenin, SNAI3 and SMAD1. Reduction of this clusters of EMT markers exhibited a halt in GBM U-87 aggression.

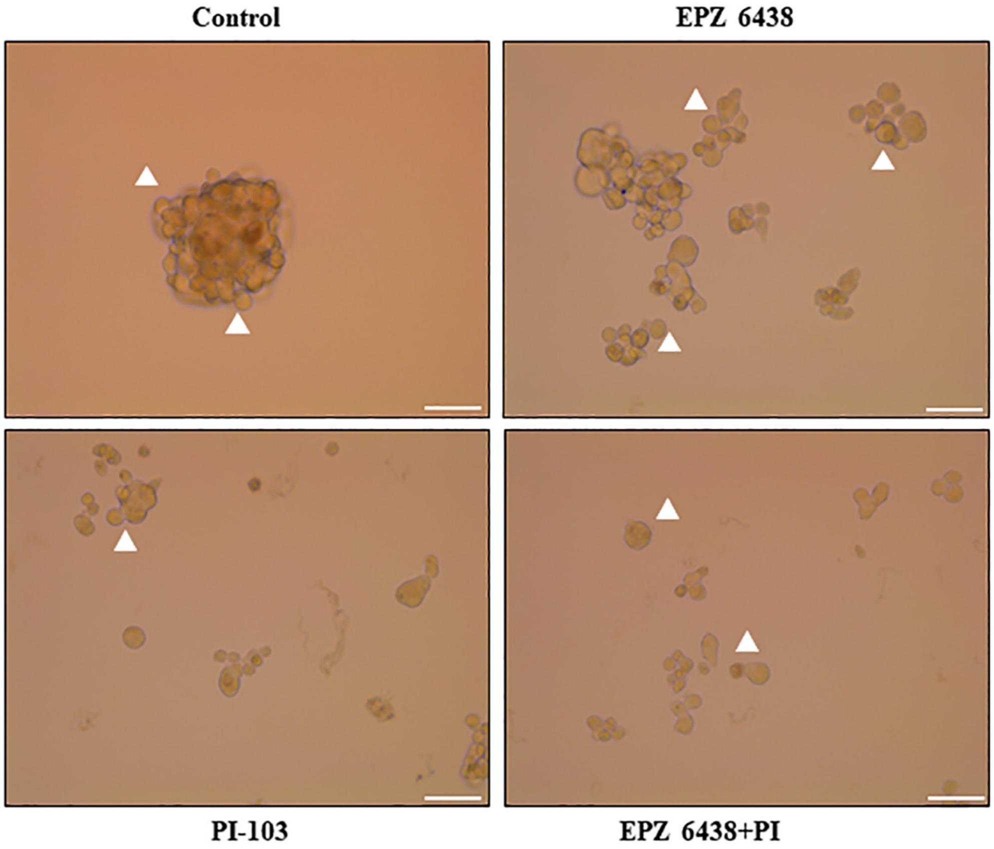

Supplementary Figure 3: EPZ-6438 and PI-103 combination affect the stemness properties of GBM U-87 cells. Combination of EPZ-6438 and PI-103 effectively reduced spheroid formation compared to control cells. Scale bar is 100 μM.

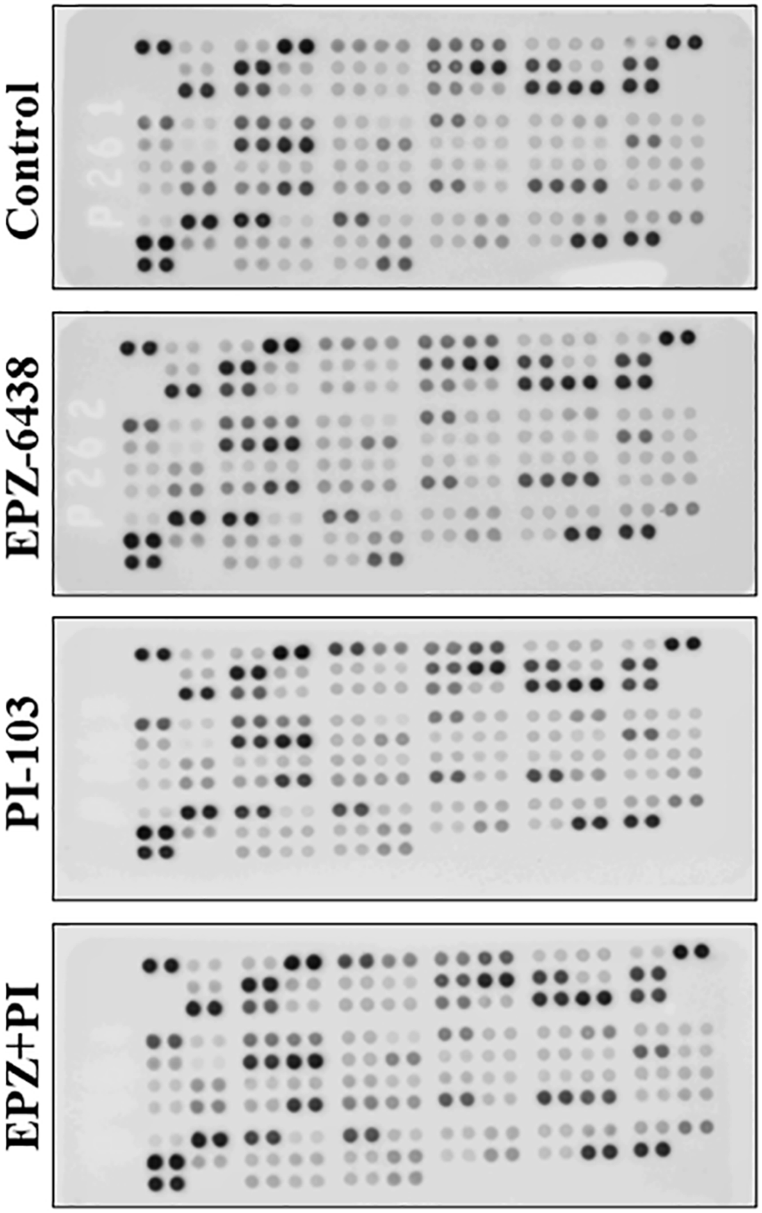

Supplementary Figure 4: Cytokine array analysis. Representation of all the membranes utilized in the experiments with different treatment. Heat Map depicts the expression level of all the affected proteins in Figure 6A.
